# Approaches of dengue control: vaccine strategies and future aspects

**DOI:** 10.3389/fimmu.2024.1362780

**Published:** 2024-02-29

**Authors:** Runa Akter, Faria Tasneem, Shuvo Das, Mahfuza Afroz Soma, Ilias Georgakopoulos-Soares, Rifat Tasnim Juthi, Saiful Arefeen Sazed

**Affiliations:** ^1^ Department of Pharmacy, Independent University Bangladesh, Dhaka, Bangladesh; ^2^ Department of Clinical Pharmacy and Pharmacology, Faculty of Pharmacy, University of Dhaka, Dhaka, Bangladesh; ^3^ Department of Pharmacy, University of Asia Pacific, Dhaka, Bangladesh; ^4^ Institute for Personalized Medicine, Department of Biochemistry and Molecular Biology, Pennsylvania State University College of Medicine, Hershey, PA, United States; ^5^ Department of Biochemistry and Molecular Biology, University of Dhaka, Dhaka, Bangladesh

**Keywords:** dengue, dengue vaccine, vector control, live attenuated vaccine, inactivated vaccine, recombinant vaccine, viral vector vaccine, DNA vaccine

## Abstract

Dengue, caused by the dengue virus (DENV), affects millions of people worldwide every year. This virus has two distinct life cycles, one in the human and another in the mosquito, and both cycles are crucial to be controlled. To control the vector of DENV, the mosquito *Aedes aegypti*, scientists employed many techniques, which were later proved ineffective and harmful in many ways. Consequently, the attention shifted to the development of a vaccine; researchers have targeted the E protein, a surface protein of the virus and the NS1 protein, an extracellular protein. There are several types of vaccines developed so far, such as live attenuated vaccines, recombinant subunit vaccines, inactivated virus vaccines, viral vectored vaccines, DNA vaccines, and mRNA vaccines. Along with these, scientists are exploring new strategies of developing improved version of the vaccine by employing recombinant DNA plasmid against NS1 and also aiming to prevent the infection by blocking the DENV life cycle inside the mosquitoes. Here, we discussed the aspects of research in the field of vaccines until now and identified some prospects for future vaccine developments.

## 1 History, epidemiology and etiology of dengue

The most widespread mosquito-borne disease in tropical as well as subtropical regions is dengue which affects 100 to 400 million people per year worldwide and poses a threat of dengue infection to nearly half the world’s population (1). Being the most frequent arboviral disease globally, it has already infected people across approximately 128 countries, a number that is about to increase even more in terms of incidence and geographic expansion, and is undoubtedly (2, 3) distressing international travelers (4, 5).

Southeast Asia, the Eastern Mediterranean, Africa, the Western Pacific, and the United States are among the native hotspots of DENV infections (6), however, this epidemic is emerging as an outbreak throughout the world. WHO has already declared dengue as the second most serious viral disease after COVID-19, since the number of infected cases surged in 2020 (1). The highest DENV infected cases were reported to take place in the Philippines, Vietnam, India, Colombia, and Brazil (7). Unplanned rapid urbanization along with improper vector control management are the prime causes of transmission of mosquito-borne diseases. Propagation is also accelerated via business or personal travel (8).

Originating from the Flaviviridae family, the DENV is the predominant cause of dengue fever (DF). Positive-sense RNA with a single strand makes up the DENV genome that encodes seven nonstructural proteins (NS) in addition to three structural proteins, which are the capsid protein (C), the pre-membrane protein (prM) and the envelop protein (E) (9). These are organized as 5′-UTR-C-prM-E-NS1-NS2A-NS2B-NS3-NS4A-NS4B-NS5-UTR-3′, where UTR means untranslated region. The mature virus particles are round with a diameter of 50 nm (10). The DENV E protein, which is found on the virion surface, contains the main epitopes needed to generate neutralizing antibodies. As a result, it is an ideal target for vaccine development. Additionally, the infected patient serum has high concentrations of a protein known as DENV NS1 released by affected cells of the body. As NS1 is an extracellular viral component, it could serve as another choice for a DENV infection vaccine (11, 12).

*Aedes* mosquitoes are the actual culprit for the transmission of four serotypes (DENV 1–4) in humans (9). The fifth and the latest addition to the existing serotypes of DENV is DENV-5 which was discovered in Sarawak, Malaysia in 2013 (13). In that case, DENV-4 was transmitted between non-human primates (NHP) and mosquitoes (*A. nivalis)* and was considered a sylvatic dengue infection (14). A thorough genetic verification process was done which led to a new DENV-5 serotype prevailing in Southeast Asian forests (13). Genetic conversion from sylvatic strains to human strains, higher mutation rates, and enormous deforestation are the underlying possible factors that lead to the upsurge in transmission. The inception of the DENV-5 serotype possessing a distinct phylogene (13) is an indication that novel DENV is evolving and will be continued by contact with the infected populations due to changes in ecosystem and climate (15).

In most of the cases, dengue infections are asymptomatic/subclinical (16). Primary symptomatic infection with any serotype is manifested as an acute febrile illness that cannot be distinguished from other febrile illnesses. Fever, headache, persistent pain in the eyelid, severe muscular and joint ache, abdominal pain, flushing, and anorexia are a few typical symptoms. Any of the dengue serotypes can induce DF following dengue hemorrhagic fever (DHF), and sometimes, dengue shock syndrome (DSS) (17). The most severe effects, DHF and DSS, are more frequently observed in infections of children and teenagers under the age of fifteen (17, 18).

DENV infection develops high neutralizing antibody titers comprising potential elements of the protective immune response (19, 20). It can provide long-term effectiveness in terms of homotypic protection, but efficacy lasts only for two years in the case of heterotypic protection (21). In addition, the cross-antibody titer concentration drops and the following heterotypic dengue infection becomes more severe than the initial infection (22). Non-neutralizing antibodies are the cause of the hazardous phenomena known as antibody-dependent enhancement (ADE). The ADE often occurs when non-neutralizing antibody causes enhanced entry of virus into the host cell leading to elevated infection. They combine with DENV particles to create complexes that facilitate infection of phagocytic cells through Fc receptors. This is particularly common when previous infection with a serotype leads to enhanced entry of other different serotypes. This enhanced infection results in DHF and DSS (17).

### 1.1 Life cycle of dengue between humans and mosquitoes

**Figure 1:**
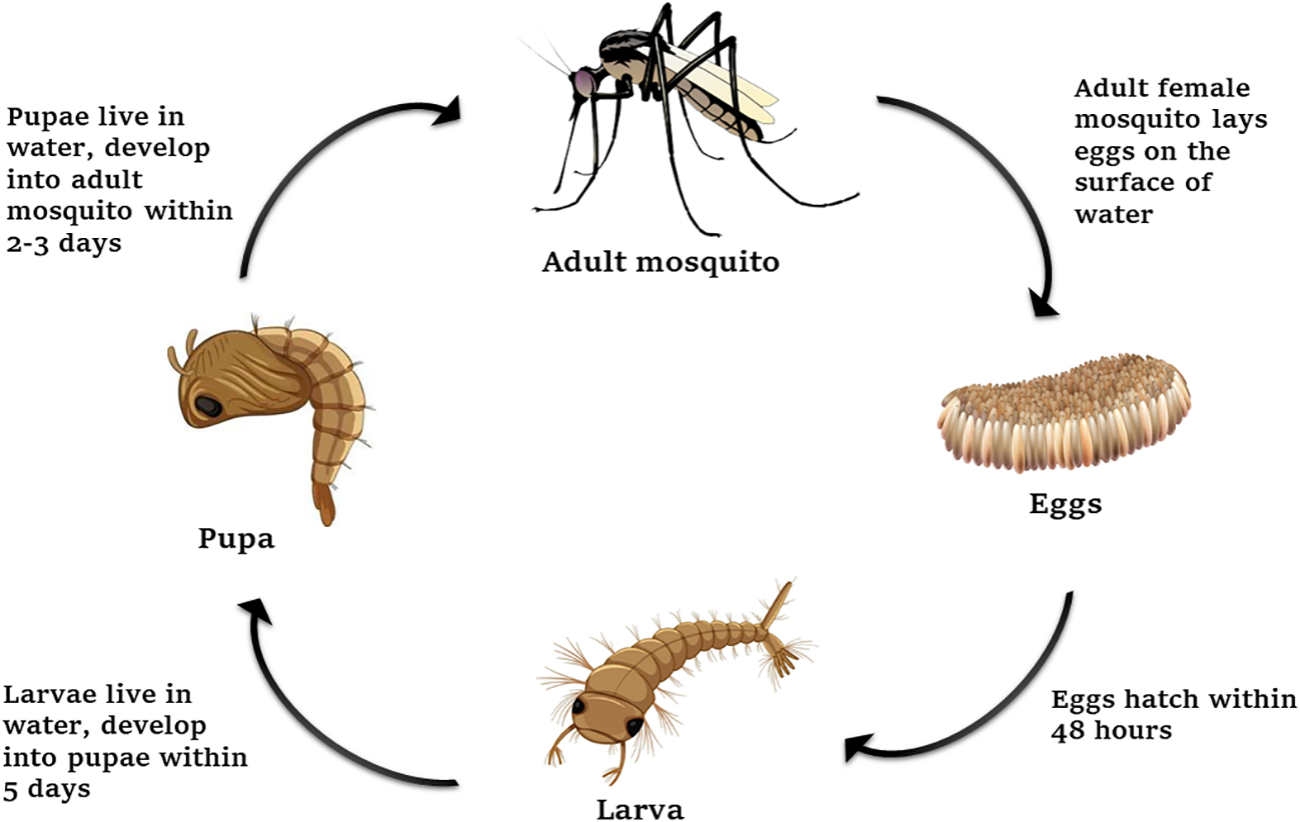
Life cycle of *Aedes* mosquitoes.

Before discussing the approaches to control dengue, we will delve into the lifecycle of dengue (**Figure 1**) to understand how the vector was aimed to be controlled. There are two different life cycles for DENV transmission among mosquitoes, primates, and humans - the sylvatic cycle and the urban cycle (23). Lower primates and humans are the only mammalian targets for dengue channeling. DENV-1 to DENV-4, all four serotypes cause infections in the urban cycle. When a mosquito bites a person who is infected with a virus, the mosquito’s midgut epithelial cells become infested with it. The virus rapidly spreads within the mosquito, moving from its hemocoel to its salivary glands and other tissues. Once this occurs, the mosquito becomes capable of transmitting the virus to uninfected humans through its bite (24). Because *A. aegypti* often breeds near human dwellings and is vulnerable to DENV, it is considered the main vector in tropical and subtropical regions. *A. albopictus*, deemed as the Asian tiger mosquito, can act as a secondary vector. For being able to survive in temperate regions, it has been rapidly spreading in all continents, and causing arboviral diseases, including DENV. *A. albopictus* plays a pivotal role in dengue transmission in Europe and China (8). 

### 1.2 Approaches to dengue vector control

The development of a dengue vaccine and treatments for DHF and DSS took longer than anticipated. As a consequence, vector control continued to be essential for the prevention and management of dengue.

The most popular and easiest way to control dengue is mosquito repellents that are mixtures of synthetic or naturally occurring compounds. The main ingredient is N, N-diethyl-3-methylbenzamide (DEET) which is very effective against mosquitoes. But continuous exposure of it can cause toxic reactions to human body (25).

One of the earliest chemical control methods used to target the adult phases of the dengue vector was dichlorodiphenyltrichloroethane (DDT). Although substantial declines in vector populations were achieved, one of the main reasons for the resurgence of dengue from the dormant stage was the onset of DDT resistance in the 1960s and subsequent years (26). Second- and third-generation insecticides (such as malathion and pyrethroids) also became accessible during this time. In addition, there are various chemical-based techniques available for controlling adult mosquitoes and the larval and pupal aquatic stages of dengue vectors. Nevertheless, chemical-based control of dengue vectors has drawbacks, such as contamination of the environment, toxin-bioaccumulation, and the plausibility of human toxicity, which are particularly linked to the application of insecticides in potable water containers (27–29).

One of the proposed strategies for vector control is biological control. This includes the introduction of larvivorous predators, such as copepods, and insect larvae into water containers, the dissemination of transgenic vectors e.g. *Wolbachia* strain (30) (intended to lessen or supplant the wild-type vector population with populations that have a limited transmission and reproduction capability), and ecological management (such as limiting the potential larva development sites, identifying and extracting abandoned containers, masking functional water containers, reduction of water pollution and ensuring the availability of safe water, reducing human-vector contact by utilizing insecticide-treated nets, and screening doors and windows). However, these alternative vector control techniques have scant evidence of their efficacy (16, 31).

Therefore, the predominant strategy to counteract dengue outbreaks has been vector control. Simply put, by lowering insect concentrations below an entomological threshold, disease transmission can be slowed. However, research on vaccines to fight dengue is also ongoing as interest in preventative measures rises. Due to the growing public awareness and the invention of novel molecular techniques, dengue vaccine development efforts have substantially accelerated during the past ten years. Some prospective candidates are being assessed from preclinical to phase three for vaccine development, such as inactivated, live attenuated, DNA, recombinant subunit, and viral vectors (32).

## 2 Dengue vaccine research and development

In 1929, the earliest dengue vaccine evaluations took place (33, 34). Vaccine formulation has been, nevertheless, hampered by several issues. First, DENV, comprising of four antigenically distinct complicated serotypes, has made vaccine development a great challenge (35). Second, DHF and DSS resulting from a second heterotypic infection with unknown immune response and pathogenesis have hindered DENV vaccine development (36, 37). Third, the adapted immunological reaction to the virus is not fully understood and the precise trait of immune responses (protective or pathogenic) to the virus is perplexing the development of a vaccine. Also, we do not have a convenient animal model that is accessible, affordable, and that accurately mirrors the immune responses in humans following infection (32). NHPs can sustain viral replication and develop a robust immune response but are unable to develop overt disease. On the other hand, certain immunodeficient mouse models infected with mouse-adapted DENV strains show signs of severe disease, like the ‘vascular-leak’ syndrome seen in severe dengue in humans. Humanized mouse models can sustain DENV replication and show some signs of disease, but to validate the immune response, further development is necessary. In the case of immunocompetent mice being infected with DENV, they do not manifest the disease. Although the prevalence of DENV-containing immune complexes in serum suggests the possibility of an ADE-like phenomenon, the swine model for DENV still has some limitations of being asymptomatic (38, 39).

Despite these obstacles, vaccine development has evolved significantly in the last few years, and the current lineup for dengue vaccines in development and testing is advanced, varied, and encouraging. Currently, multiple candidates of dengue vaccine are undergoing phase III clinical studies, and others have been evaluated in human trials (40). Various strategies for developing a dengue vaccine are addressed herein.

### 2.1 Live attenuated vaccines

The first significant attempt to create a live attenuated vaccine was made by the University of Hawaii, employing the long-established strategy of virus passage in an NHP serially. Later, the project was moved to Mahidol University (Bangkok, Thailand) for additional virus passaging, vaccine formulation, and evaluation (41, 42). Even though the proposed vaccine was unsuccessful, the study acted as a spur for later advancements toward the development of a live attenuated tetravalent dengue vaccine (43).

The Walter Reed Army Institute of Research (WRAIR) had developed the second tissue-culture-passaged dengue vaccine. The original formulation of the tetravalent dengue vaccine manufactured by WRAIR similarly showed issues with uneven reactogenicity and immunogenicity (44). In a phase II investigation, changed formulations appeared as innocuous and immunogenic; nevertheless, additional research is required to fully assess their protective effects (45).

Using the mutagenesis technique, the US National Institutes of Health, launched a novel phase of dengue vaccine research. The genetic modification of dengue genomes was simple, resulting in attenuated strains that had been evaluated on adults in the US who had not previously been exposed to flaviviruses. Additionally, a molecularly attenuated, tetravalent dengue vaccine was developed by the US FDA and tested on primates from other species, except humans (46–48). In order to develop live attenuated tetravalent dengue vaccines, both methods offer an alternative strategy (48, 49).

A number of live attenuated dengue vaccines are being developed using recombinant DNA technology, including the tetra-live attenuated virus dengue vaccine (DENVax), the recombinant DENV-4 mutant vaccine carrying deletion of 30-nucleotide (rDEN4Δ30), and the tetravalent dengue vaccine with chimeric yellow fever 17D virus (CYD-TDV) (50, 51).

#### 2.1.1 Live attenuated chimeric yellow fever–dengue vaccines

A tetravalent chimeric dengue vaccine was designed by the US Centers for Disease Control and Prevention (CDC) by incorporating the E genes and prM of DENV-1, DENV-3, and DENV-4 into the cDNA acquired from the effectively attenuated DENV-2 unit of the Mahidol University-Sanofi Pasteur live attenuated DENV vaccine (DEN-2, 16681 PDK-53) (49, 52). The infective cDNA component of the prominent yellow fever vaccine virus, strain 17D was modified to incorporate dengue structural genes. Beginnings for this can be found in the medical schools of Washington and St. Louis Universities. Sanofi Pasteur has been granted a manufacturing license by Acambis, Inc. to continue the commercial development of these yellow fever chimeras (53–55).

**Figure 2:**
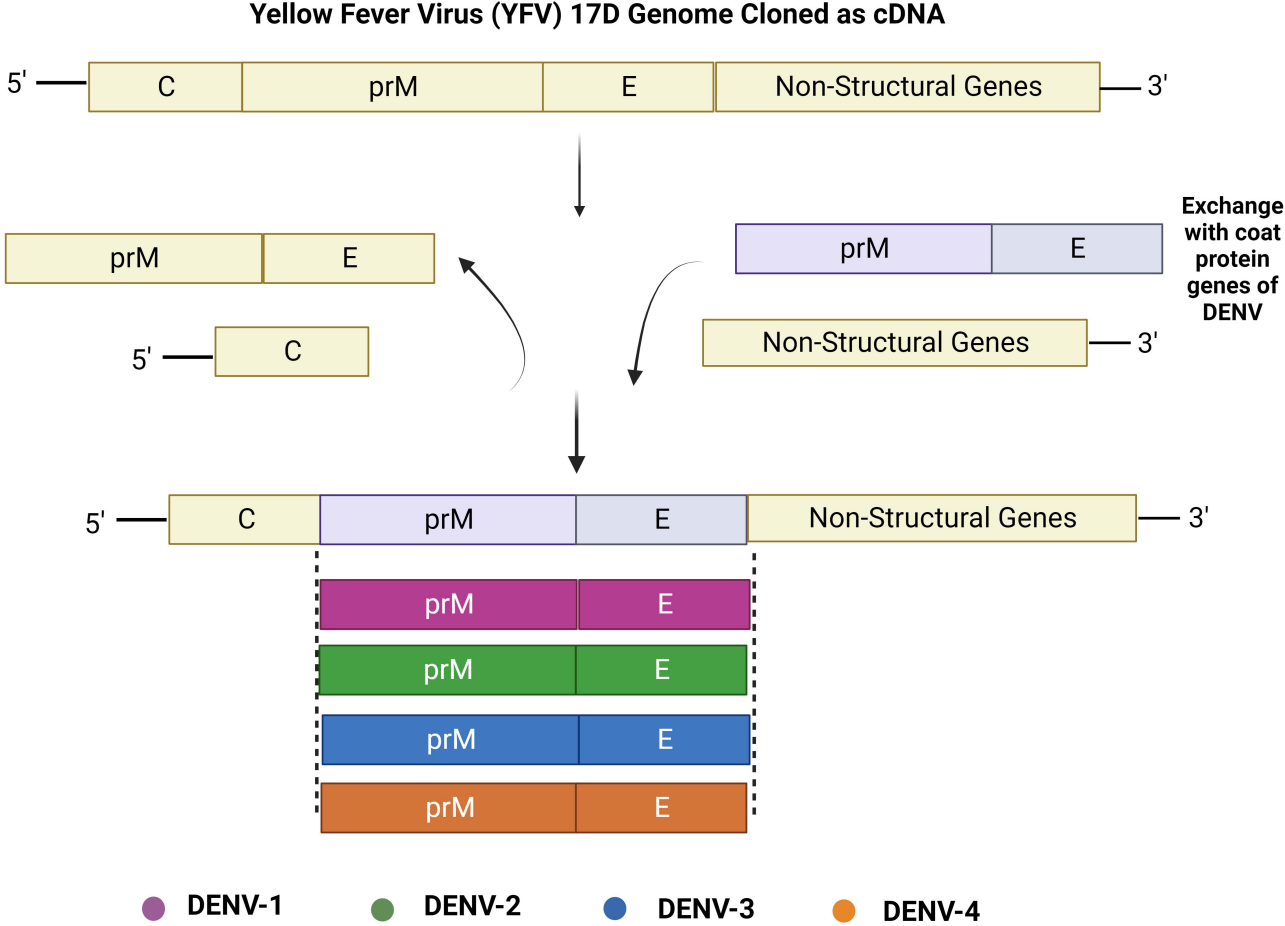
Construction of live attenuated chimeric yellow fever–dengue vaccines. Genes encoding for prM and E proteins from the cDNA backbone of yellow fever vaccine virus strain 17D are substituted with those of heterologous DENV serotypes: DENV-1, DENV-2, DENV-3, and DENV-4 to create these chimeric flavivirus vaccines.

In consequence, Sanofi Pasteur (Lyon, France) developed the foremost authorized dengue vaccine named Dengvaxia. Using Vero cells as a substrate, this dengue vaccine is a tetravalent chimeric which has received marketing authorization for clinical studies in Mexico and the Philippines (56, 57). The four dengue serotypes’ matching sequences were substituted for the YF17D yellow fever virus prM/E RNAs to develop the respective vaccines (**Figure 2**) (58, 59). This putative vaccine was immune-modulating for all the DENV serotypes and may have provided protection against serotypes 1, 3, and 4 (60).

In Thailand, a phase IIb investigation conducted on this particular vaccine reported high efficacy against DENV-3 and DENV-4 serotypes, moderate activity against DENV-1, and no potential to counter DENV-2 infection (55, 60, 61). Additionally, two phase III trials were carried out in the dengue-endemic Asia-Pacific region and five nations in Latin America. The results clearly demonstrated that the vaccines’ efficacy against DENV-1, DENV-2, DENV-3, and DENV-4 was 50.3%, 42.3%, 74.0%, and 77.7% in Latin America (62), and that its average efficacy against dengue-associated clinical indications and hospitalization was 56.5% in Asia-Pacific (63). These data demand further evaluation of the efficiency and protection by the Dengvaxia vaccine (64).

Immunity stimulated by the vaccine lasts up to four years, but the efficacy is affected by many factors such as virus serotype, age, and individual dengue sera status (65). It is reported that seropositive subjects aging over nine years gain conclusive protectivity by CYD-TDV. According to the recommendation of the WHO Strategic Advisory Panel, seronegative patients should not be vaccinated as the risk of severe dengue is increased via CYD-TDV vaccination (66). For the assessment of serum condition, the plaque reduction neutralization test, recognized as the gold standard, necessitates distinct technical and laboratory facilities.

Over conventional attenuation in cell culture, molecular clone-supported approaches for a tetravalent dengue vaccination provide significant benefits. These include a molecular explanation for attenuation and lessening the threat of inadvertent agents, both of which will lower the costs associated with product quality assurance.

#### 2.1.2 Live attenuated rDENΔ30 vaccines

Remarkable studies based on the integration of the live attenuated tetravalent vaccine (LATV) with DENV monovalent vaccines have been conducted (67). Since the 3’-UTR of the genome of flavivirus is crucial for the RNA replication, it has been selected as the focal point (68, 69).

According to Durbin, on the 28^th^ day after vaccination, a boost in serum neutralizing antibody titer has been increased to a seven fold or more (mean titer = 1:580), which was noted to have satisfactory tolerance (70). Obtaining rDEN-4Δ30 NS3-S158R through chemical mutagenesis from rDEN4Δ30 was a huge success (71–73).

Conversely, the TV003 vaccine, which combines four attenuated recombinant dengue vaccines (rDEN1Δ30, rDEN2/4Δ30, rDEN3Δ30/31, and rDEN4Δ30), exhibits higher resistance against DENV-2 compared to CYD-TDV (74). TV003 was reported to produce antibodies against each of the DENV serotypes among 91.7% of trial-subjects, and the potentiality to protect opposing DENV-2 was higher than CYD-TDV vaccination manifesting mild rash only as an adverse reaction (75). A comparative phase I clinical trial showed TV005 was combined with more DENV-2 attenuated virus components than TV003. A comparatively balanced immune response was produced in 90% of vaccines by a single vaccination of TV005, whereas TV003 vaccination produced an immune response in 76% of subjects only (76). TV003 and TV005 stimulate the proportionate production of neutralizing antibodies compared to other LATVs (TV001–TV005) since these vaccines have diverse virus particle structures, infectivity, and immunogenicity (77). Nonetheless, it can be challenging to generate a LATV that demonstrates an immunogenic response against every serotype and attenuates each monovalent component at the same time (46, 78, 79).

#### 2.1.3 Live attenuated chimeric tetra-dengue vaccines

For producing the live attenuated chimeric tetra-dengue vaccines, at first the genetic code of the DENV-2, PDK-53 was altered and coding sequences of DENV-1, DENV- 3, and DENV-4 were included. Finally, by transferring the recombinant RNAs into Vero cells, the potential candidate DENVax was produced (80). Currently, these vaccines are undergoing clinical trials. However, research in AG129 mice indicates that the immunization effects on offspring, born by PDK53 immunized mothers, may be interfered due to maternal antibodies. Live attenuated vaccine contains definitive protectivity for the progeny over 9 years old. Due to high immunogenetic tolerance of DENVax, it is quite challenging to elicit systemic reactions from it (50).

### 2.2 Inactivated virus vaccines

**Figure 3:**
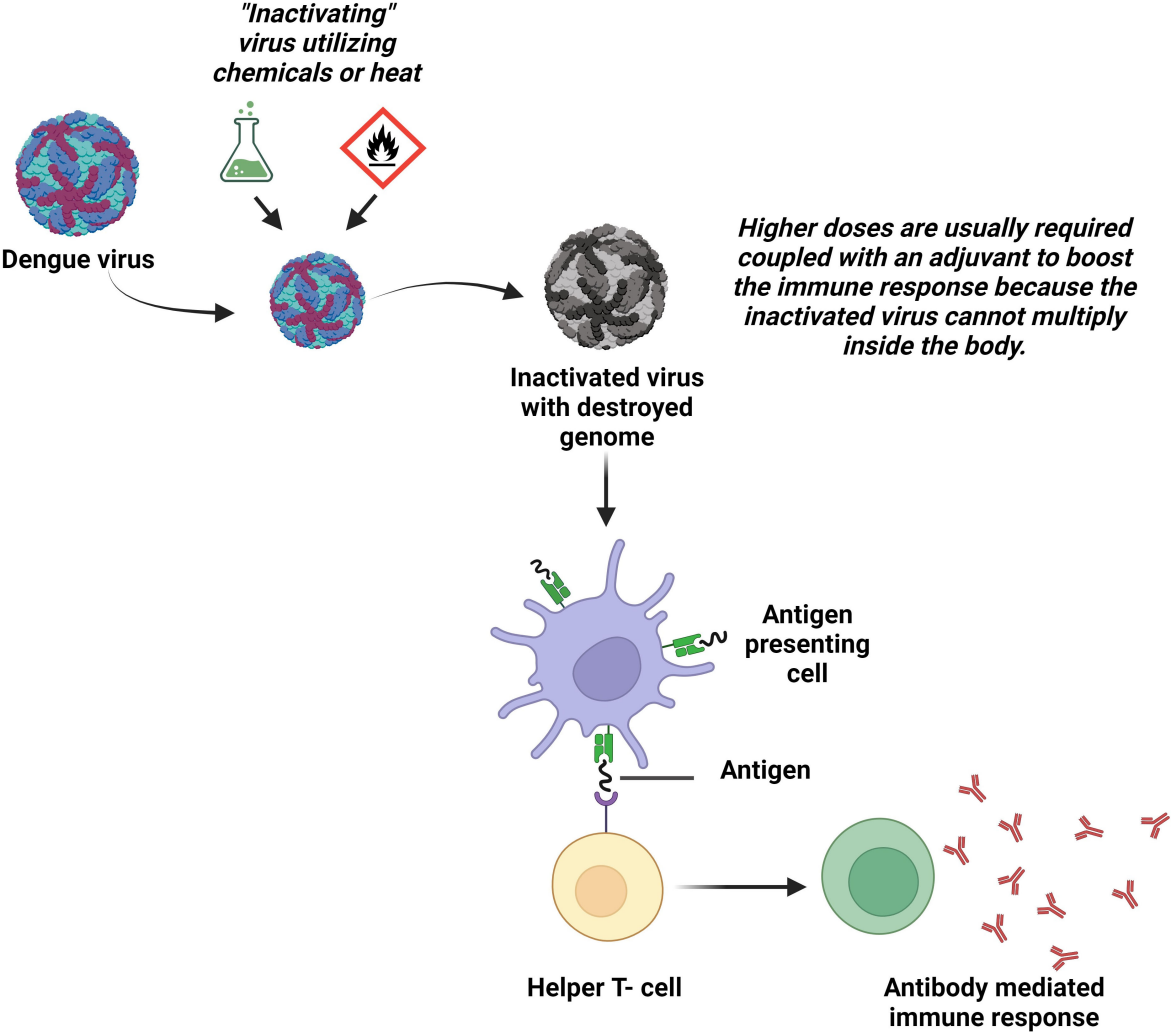
Inactivated virus vaccines. The wild DENV is rendered incapable of reproducing or causing diseases in the body by applying heat or chemicals in the laboratory. The body produces antibodies in response to the vaccinations made from these inactivated viruses.

Another way for the protection from live pathogens could be inactivated vaccines that comprise inactivated material derived from viruses or bacteria (**Figure 3**). S16803, which was produced utilizing formalin-inactivation and sucrose-centrifugal processes, is an illustration of a DENV-2-inactivated vaccine (81). Immunogenicity was assessed in *Macaca mulatta* using three different types of vaccines: a recombinant subunit protein vaccine (R80E), a live attenuated vaccine (DENV-2 PDK-50), and an inactivated vaccine (S16803). The result showed that stable titers of antibodies were only produced by DENV-2 PDK-50 (82). Initial vaccination of rhesus monkeys with tetravalent purified formalin-inactivated vaccine (TPIV) or tetravalent DNA vaccine (TVDV) followed by a tetravalent live attenuated vaccine (TLAV) revealed stimulated humoral immune responses against DENV in comparison to immunization with individual vaccine type (83, 84). The underlying mechanism is: initially TPIV helps reach all the titers of four serotypes of antibodies to a definite height and then immunization is enhanced by TLAV (84, 85).

Applying inactivated virus vaccines is quite challenging. This is because, these vaccines only express the part of the viral genome responsible for structural proteins, leaving them unable to stimulate immunity against the NS proteins. For gaining optimal immunogenicity in seronegative subjects, the addition of adjuvants may be an option, which in turn could increase the cost and reactogenicity of the vaccine. Another disadvantage is that, to ensure long-term immunity, multiple booster doses are required. In contrast, since they cannot change into a more pathogenic phenotype and are less likely to interact when combined, inactivated whole virus vaccines are advantageous. Additionally, it has been shown that inactivated flavivirus vaccines can induce both cell-mediated and humoral immune responses (86).

### 2.3 Recombinant subunit vaccines

**Figure 4:**
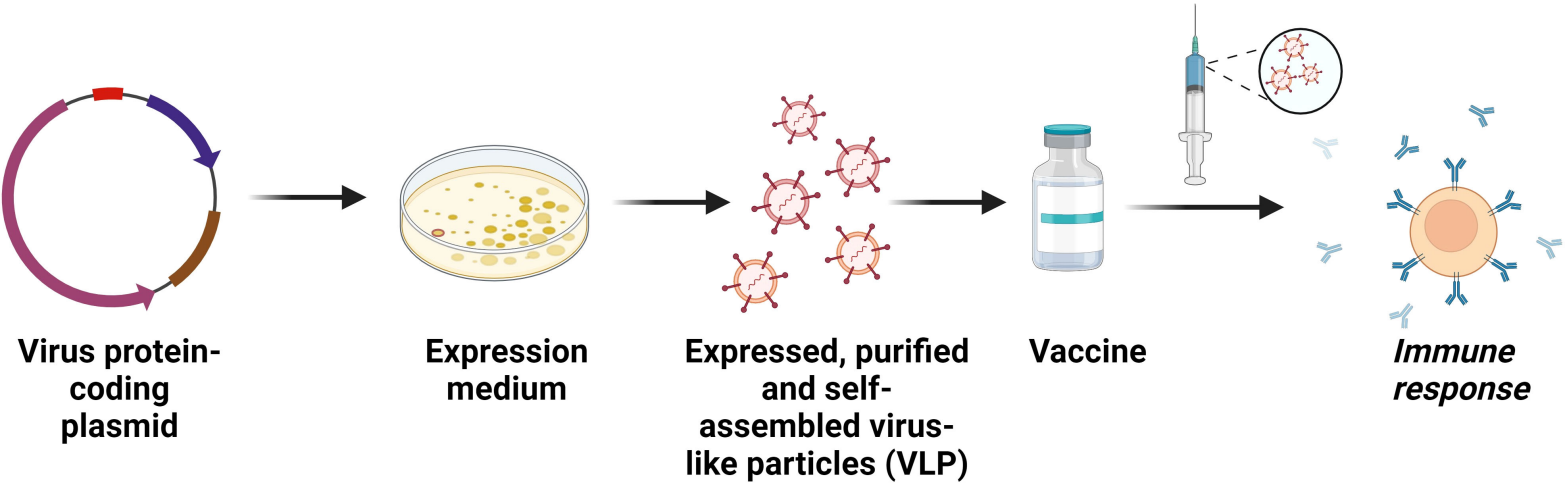
Recombinant subunit vaccines. Construction of subunit vaccines comprising purified viral proteins that act as antigens to induce immunity in host. A modified form of the subunit vaccine is the viral-like particles (VLP) vaccine. VLPs are entities made up of one or more proteins that resemble real viruses but do not have a viral genome. Effective immunological responses from T cells and B cells have been observed following the administration of these vaccines.

A variety of expression techniques have been used to manufacture dengue antigens, especially E proteins, to create candidates for subunit vaccines (87). Eukaryotic or prokaryotic cells frequently express these antigenic proteins to elicit durable defensive or curative immune responses (**Figure 4**) (88).

It was demonstrated that the recombinant envelope protein domain III (EDIII) from *E. coli* efficiently induces the production of antibodies against all the dengue serotypes in mice (89). Moreover, the protection of lactating mice from infection by these antibodies was also reported (90). A lipoprotein in combination with recombinant dengue proteins eliminated the use of adjuvants and immune responses against all the serotypes were provoked substantially. Here, lipoprotein is the main element of intact bacteria that activates monocytes through a CD14-dependent route in spirochaete and plays a significant role in determining the spirochaete’s pro-inflammatory potential (91–93).

One tetravalent dengue vaccine, called EDIII-P64K, contains adjuvant that expresses EDIII from various DENV serotypes and P64K from *Neisseria meningitidis*. Mice that received the vaccine three times, produced high levels of DENV1-3 and low levels of DENV-4 antibodies (94). Additionally, using *E. coli* to express DENV1–2 EDIII was combined with DENV3–4 EDIII via Gly-Ser linker, and mice receiving this combination of vaccinations successfully developed a defense against all serotypes of DENV (95, 96).

By using heat labile enterotoxin B subunit (LTB) derived from *E. coli* and synthetic consensus dengue envelope domain III (scEDIII), fusion protein LTB-scEDIII was produced. By immunizing with cell-free extracts (CFE) and recombinant yeast cells (rYC) via oral route, mucosal and systemic humoral immune responses have been observed. Concentration of neutralizing antibodies was higher in case of CFE rather than rYC (97).

V180 is considered another potential subunit vaccine made of insect cell-derived truncated protein DEN-80E. Vaccination of mice and rhesus monkeys with this showed remarkable immune protection against DENV with a low dose only (50, 98). On top of that, V180-immunization can protect rhesus monkeys from viremia (99).

A study showed that transgenic plants and ovarian cells of Chinese hamster were used to produce a protein containing polymeric immunoglobulin G scaffold and DENV consensus domain III (cEDIII). That protein induced large production of IgG antibodies in the mice, possessing DENV-2 neutralizing capacity (100).

The advantage of recombinant subunit vaccines is that they can induce balanced immunologic responses against all the DENV serotypes as well as reduce the incidence of ADE effect in comparison with live attenuated vaccines. A lack of post-translational protein processing, however, could initiate protein generation that are different from inherent responses of antibody and other proteins (101, 102).

### 2.4 Viral vector vaccines

**Figure 5:**
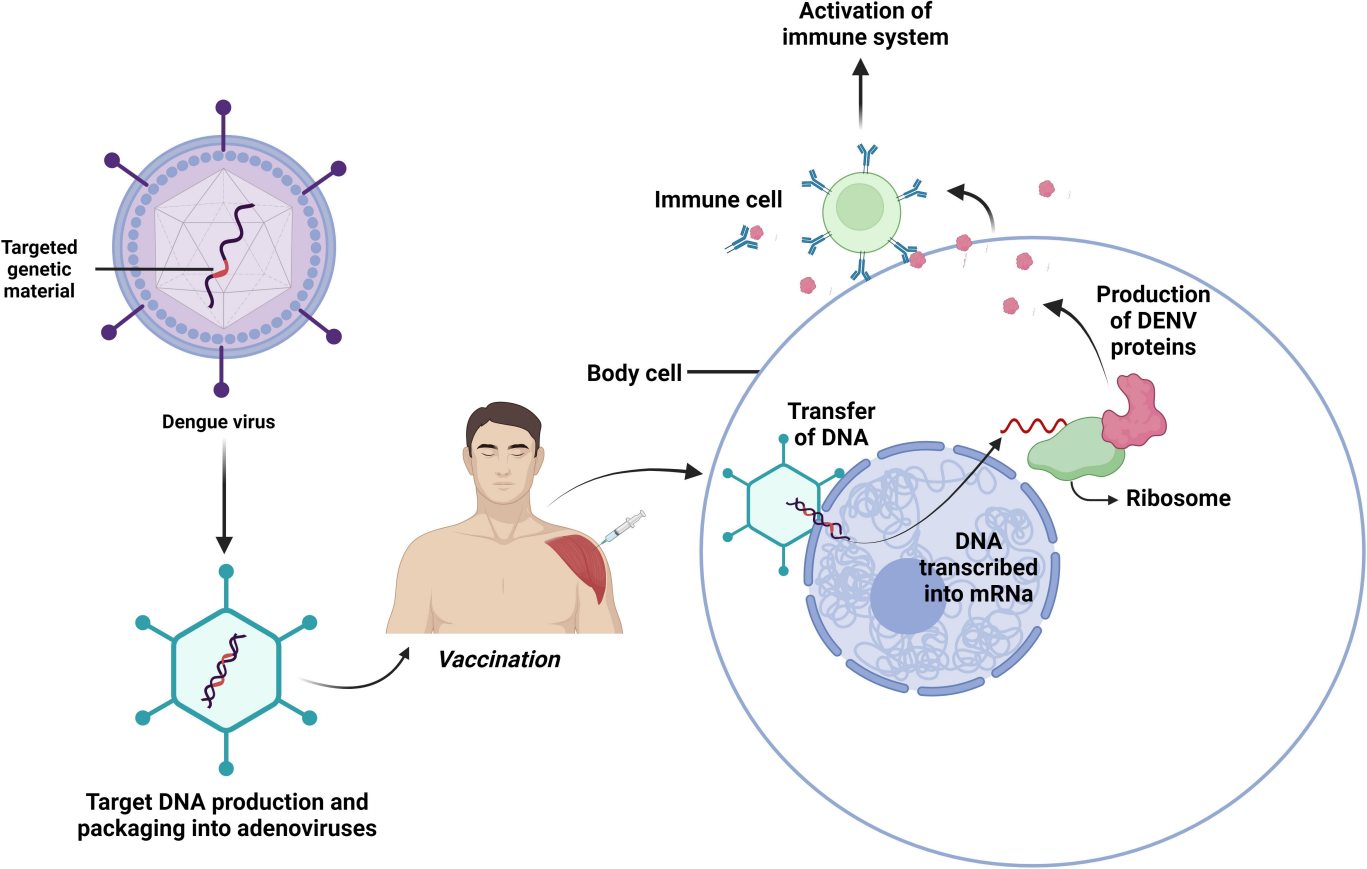
Viral vectored vaccines. Composed of a brief segment of a virus’s genetic material that contains the instructions for the target protein. Another virus that has undergone genetic modification so it cannot replicate itself in the human body delivers the gene safely to host cells to develop immunity against invaders.

Adenoviruses, alphaviruses, and the Vaccinia virus are some common vectors used for delivering dengue viral antigens in the formulation of vaccines (**Figure 5**) (103). Since the efficiency of DENV-4 proteins (prM, E, NS1, and NS2A) in the Cidofovir-resistant vaccinia (WR) strain was not up to the mark (103, 104), in order to increase protectivity, whole vaccinia virus or c-terminal truncated ones were recombined to generate the DENV E protein (50). MVA-DENV2-80%E and MVA-DENV4-80%E were created based on the innocuous, altered vaccinia Ankara (MVA) virus. Although the early viruses only mildly induced DENV-2 antibodies in rhesus monkeys, these viruses can produce large anti-E antibodies in mice models (105).

In the development of recombinant replication-defective adenovirus (rAd), some factors such as- easy gene manipulation, high level of protein expression, and convenient identification of gene replication flaws were essential. The vaccine was administered to the mice intra-peritoneally and successful stimulation of DENV-2 antibodies and specified T-cell immunity via the E protein of DENV-2 had been witnessed (106, 107). Additionally, mice which were initially injected with rAd and subsequently given a boost with a DNA vaccine that expressed EDIII, displayed defense against DENV-2 and DENV-4 infection (108). Another potential agent is a divalent complex Adenovirus (cAd)-vectored vaccine e.g. cAdVaxD (1–2) and cAdVaxD (3–4). The rhesus monkeys developed antibodies against all serotypes of DENV after receiving prM and E of the DENVs, as well as a T-cell immunological response (109).

Venezuelan equine encephalitis virus (VEEV) replicon particles (VRP) are alphavirus-vectored vaccines against DENV that express large amounts of antigens in a single dose of vaccination (110, 111). The VRP which expresses DENV-1 proteins such as M and E, induced defensive antibodies in cynomolgus monkeys (112). On the other hand, DENV-2 VRP produced IgG and neutralized antibodies against DENV-2. Though both prM-E-VRP and E85-VRP were capable of producing antibodies against specific serotype in rhesus monkeys, against EDIII, the E85-VRP produced antibodies more rapidly and with higher concentration (113). Given that it gave monkeys a balanced immune response and made them safe to DENV1-4, the tetravalent E85-VRP dengue vaccine can be an important tool for overcoming serotype interference. Only after a single neonatal dose, the VRP vaccine developed robust immunity, though the magnitude was not even that of adult mice (114).

The viral vector vaccine is believed to have the greatest impact on cellular immunity and is likely to elicit greater humoral responses. In contrast to different viral vector vaccines, adenoviral vectors are better for simple genetic alterations, identifying abnormalities during replication, as well as elevated antigen expression.

### 2.5 DNA vaccines

**Figure 6:**
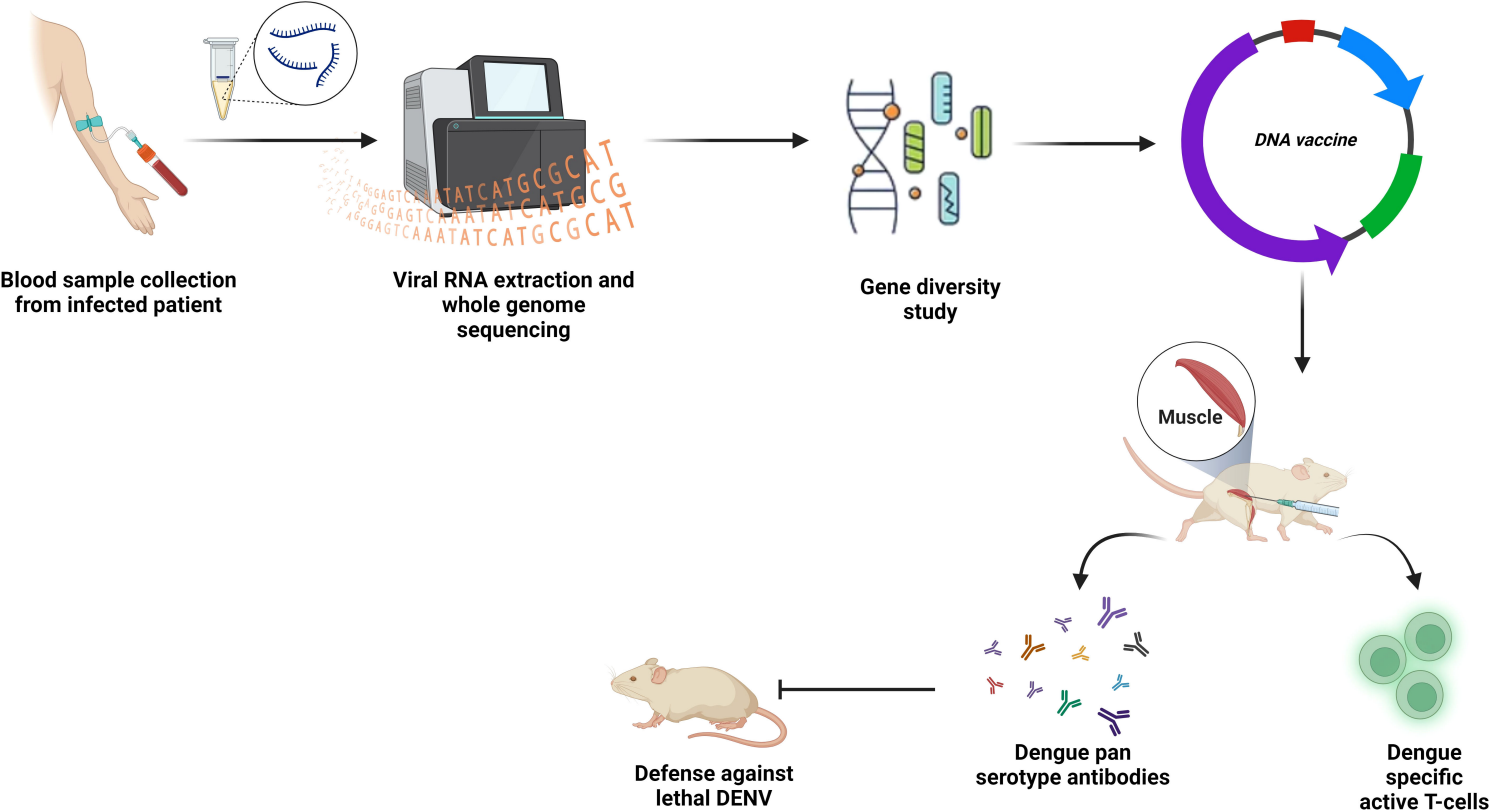
DNA vaccines. PCR, or synthetic generation, is used to obtain the desired gene sequence. To promote T cell and B cell mediated immunity in the host body, this sequence is introduced enzymatically into the multi-cloning region of a plasmid backbone, purified, and then administered at the inoculation site.

DNA vaccines can be defined as a plasmid encoding specific antigens which, if injected into living organisms, express antigens and that is how trigger immune response (115). Neutralizing antibodies were successfully developed in mice and monkeys after receiving the TVDV. Injecting BALB/c mice with an intradermal DNA vaccine including structural prM and 92% of the DENV-2 E protein, resulted in the predicted antibodies (**Figure 6**) (115). The DNA vaccine that expressed prM and 100% of the E protein (ME100) induced antibodies more potently than one that expressed only 80% of the E protein (E80) (116). Safety and well-tolerance of D1ME100 had been proven in the first phase of vaccination with little discomfort or soreness at the injection site as side effects in human trials. Only 41.6% of vaccines in the high-dose group are reported to produce neutralizing antibodies with zero response in the low-dose group (117).

According to Porter et al, DENV-2 prM/E along with CpG motif is better in defense than DENV-2 prM/E alone (118).

It is also reported that a recombined DENV-2 EDIII and *E. coli* maltose binding protein (MBP), expressed by DENV-2 DNA vaccine was responsible for triggering the generation of neutralizing antibodies (119). A comparative study using DENV-2 prM/E DNA vaccine (D), DENV-2 EDIII and MBP recombinant fusion protein (R), and purified inactivated virus particles (P) on rhesus monkeys demonstrated the higher antibody level and higher neutralization, when administered as combined rather than alone. The upmost antibody titers were observed in DR/DR/DR, DP/DP/DP, and R/R/R vaccination, whereas D/D/D vaccination showed the least. However, animals vaccinated with only P showed substantial immune protection against viremia (120).

The use of myriads of guiding proteins that target the immune system, augments vaccine protection. One of the ways of developing the immunogenic response against DENV-2 prM/E DNA vaccine is ensuring increased synthesis of T-cells through incorporation of the antigen sequence into the lysosomal membrane protein, together with anti-CD4 antigens, which results in the enhanced production of MHC class II antigens (121). Another method for inducing robust immunity to a target antigen is through the fusion of the antigen with a single-chain Fv antibody (scFv) that is selective for the DC endocytic receptor (DEC205) (122).

To assess the efficacy of the TVDV, TPIV and TLAV immunization, Rhesus monkeys were used. Only TVDV/TVDV/TLAV treated monkeys were marginally protected, whereas monkeys inoculated with TRIV/TLAV were fully viremia free (123). It has been proved by a phase I clinical study that concurrent administration of TVDV with an adjuvant called Vaxfectin^®^ (Vical, Boulder, CO, USA) induces anti-dengue T-cell IFN response safely (124).

While DNA vaccines offer advantages such as stability, easy preparation, cost-effectiveness, and apt for mass production, they require enhanced immunogenicity. Fortunately, the problem can be addressed by taking measures such as plasmid modification using alternative delivery strategies, promising promoters, multiple dosing systems, and co-immunization with adjuvants (125).

## 3 Newer strategies to immunize against dengue

Although numerous DENV vaccines have been approved or are currently undergoing clinical trials that target either the viral structural proteins or the DENV virions, there are a plethora of safety concerns that still need to be addressed. Conventional methods for developing an unerring dengue vaccine have been challenged by several issues relating to dengue pathogenesis, including the danger of ADE effects and pathogenicity entailed by cross-reactive T-cells throughout the disease progression. Having stated that, in addition to conventional approaches employed for dengue vaccine development, several unique aspects of DENV multiplication and life cycle may serve as the focal point for cutting-edge vaccine formulations.

In order to evade dengue disease, novel concepts like DENV NS1 based vaccines and mosquito based immunization approaches should be undertaken as prospective options (126). In addition to these, mRNA vaccines could be a promising choice, given the success of the mRNA COVID-19 vaccine during the pandemic.

### 3.1 Strategies based on NS1 proteins

The NS1 glycoprotein is composed of a hexamer alongside a dimer, which is the membrane-anchored type of glycosylphosphatidylinositol (127). The release of the NS1 protein in the sera of infected subjects usually ranges from 70 to 15,000 ng/mL, while in extraordinary circumstances, it reaches 50,000 ng/mL (11, 12). As an extracellular component, we can utilize NS1 as a potential vaccine candidate. According to Henchal, passive immunization against DENV in mice has been achieved via anti-NS1 antibodies (128). On the other hand, it is reported that, cranially challenged mice were partially protected by immunization with recombinant NS1 protein via the active intraperitoneal or subcutaneous route (129, 130). Two studies reported that NS1 completely prevented dengue-mediated vascular leakage while acting as a key factor in enhancing the permeability of blood vessels (37, 131). Furthermore, the mice which were deficient in interferon receptors, were protected from the fatal effects of DENV-2 (131). A promising alternative to NS1 immunization is to immunize using a protein combination consisting of protein E of DENV-2, N-terminal of NS1, along with protein A of *Staphylococcus aureus*, which helps protect the mice potentially by the generation of anti-DENV-2 antibodies (132).

NS1-based vaccines can be developed by using recombinant DNA plasmids. These DNA plasmids, by producing cytokines, DENV prM, NS1, E, and rat granulocyte-macrophage colony-stimulation factors, were reported to protect the mice from a fatal intracerebral DENV-2 challenge (133). Costa et al. also reported that, mice inoculated intracerebrally with DENV-2 NS1-consisting recombinant plasmid pcTRANS1 showed promising outcomes (134). Another study carried out in mice displayed NS1-specific humoral and cellular immunological reactions, resulting in retardation in the development of paralysis as well as a considerably extended survival after an intravenous challenge with fatal DENV-2 (135).

While recombinant vaccine viruses encoding DENV-2 or DENV-4 NS1 offer partial protection to BALB/c mice (136), several studies have suggested that NS1 antibodies may exacerbate dengue pathogenesis. This is due to their cross-reactivity with host proteins, leading to inhibited apoptosis of endothelial cells and triggering platelet aggregation (137). Further safety and effectiveness testing of the NS1 vaccination is required considering these issues.

### 3.2 Strategies based on blocking of transmission of infection

To address the spread of dengue, we must urgently implement all essential measures. For that, the number of infected vectors needs to be decreased by host specific vaccination. Vaccines must be designed such that they target the life cycles of pathogens in the environment to control the disease. By focusing on the mechanisms pathogens use to invade both vertebrate and invertebrate hosts, we can obstruct their pathways (126). For example, gametocytes of the *Plasmodium* species can be targeted to reduce the number of pathogens persisting in the mosquito gut, which in turn impairs the efficiency of microbial transmission efficiency (138–140). Immunizing against specific mosquito C-type lectins (mosGCTLs), which meet the DENV-2 virus and infect *A. aegypti*, it is possible to halt the life cycle of DENV. Research also indicates that a combination of antisera against several mosGCTLs can significantly decrease dengue infection post blood meal (141). Another study found that blocking of cysteine-rich venom protein (CRVP379) by either specific antisera or RNAi, retarded the DENV contamination in *A. aegypti*, which can be considered a promising prophylactic and curative action for controlling dengue disease (142). Combining immunization with DENV-related mosquito vulnerability variables may be a plausible concept for the development of a vaccine that prevents dengue transmission.

### 3.3 Advancements in dengue mRNA vaccine development

**Figure 7:**
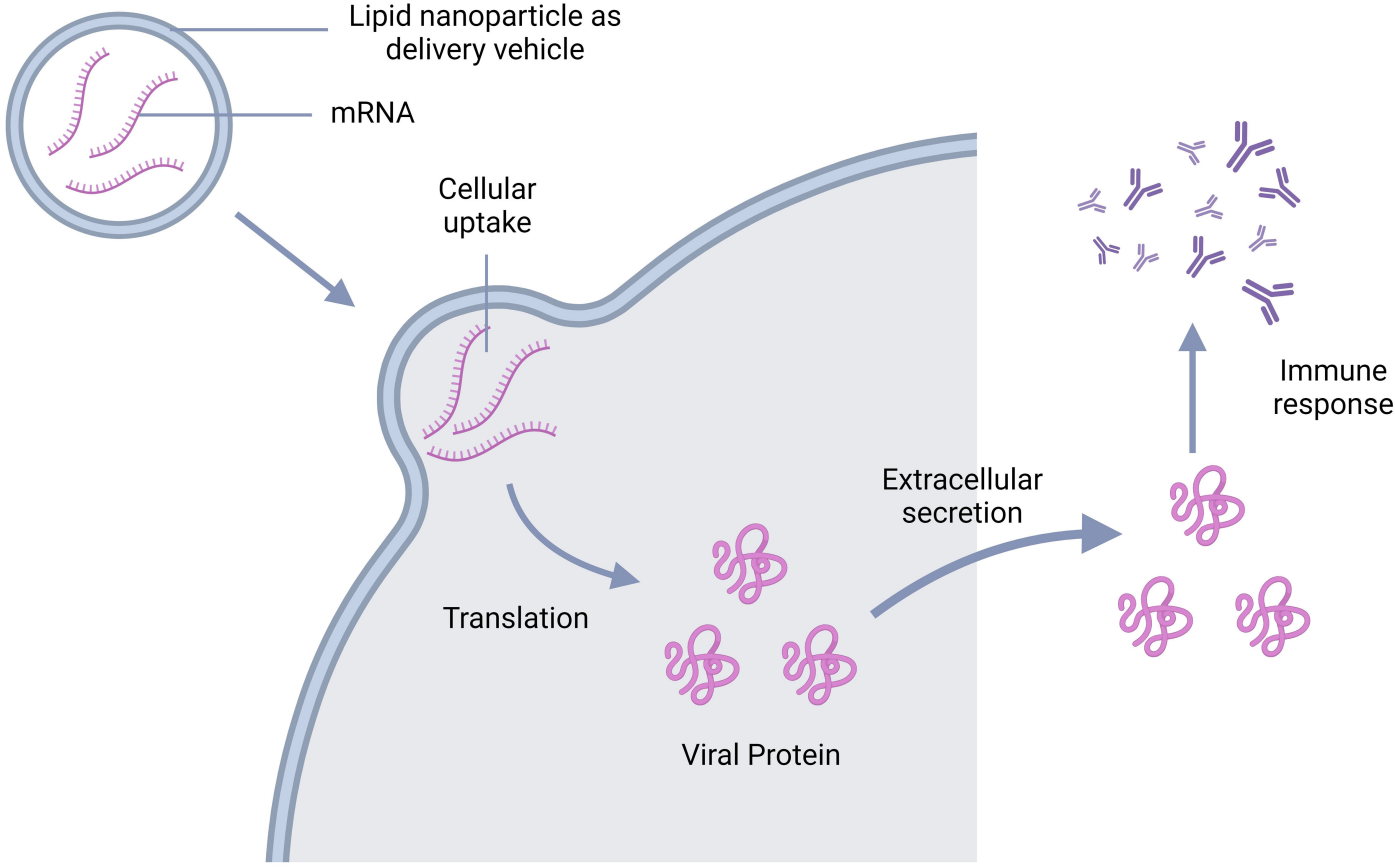
Schematic representation of the mechanism of action of mRNA vaccine; mRNA goes inside the cell leaving the vehicle outside and get translated into viral antigen. The antigens are then secreted extracellularly triggering the antibody production.

To develop an mRNA vaccine, scientists used to modify the natural structure of mRNA. The team of Wollner Clayton has pursued the same approach for the development of a dengue vaccine (**Figure 7**). They first transcribed DENV-1 membrane and envelop structural protein (prM/E) into a modified mRNA using pseudo uridine and then packed it into lipid nanoparticles (LNP). The mRNA-LNP complex was then injected intramuscularly and after the endocytosis by muscle cells, the LNP was degraded, leaving the mRNA for further translation of viral prM/E proteins. The viral polyproteins then exerted serotype-specific immune response in the study mice (143). Another group targeted prME, E80 and NS1 of DENV-2 using the same mRNA vaccine technology mentioned. In this case, they modified mRNA nucleoside to 1-methylpseudouridine-50-triphosphate (1 mψ). This candidate vaccine induces production of neutralizing antibodies against DENV-2 and triggers a T-cell immune response in mice (144).

Both aforementioned vaccines are currently in initial stages, but hold promise for approval in upcoming clinical trials. Following table (**Table 1**) summarizes the stages of development of the dengue vaccines.

**Table 1:** Summary of dengue vaccine development strategies and current status.

Name of vaccine	Producer	Type of valence	Composition	Evaluation	Adjuvanted	Ref
DENVax/TAK-003	Takeda	Tetravalent	Chimeric viruses DEN-2 PDK-53, TDV1-4	Phase III trial *in vivo*	No	(51, 145)
Dengvaxia/CYD-TDV)	Sanofi Pasteur	Tetravalent	YF17D vaccine strain as the backbone and substituting the YF17D prM and E regions with those of the four DENV serotypes	Licensed	No	(51, 145)
TV003/TV005	Tetravalent	Three genetically attenuated viruses and one chimeric virus	National Institute of Allergy and Infectious Diseases (NIAID)	*In vivo* (phase IIIB)	No	(145)
TDEN	Tetravalent	Viruses attenuated with passages in PDK cells	Walter Reed Army Institute of Research (WRAIR) and GlaxoSmithKline	*In vivo* (phase I-II)	No	(146)
DPIV	Tetravalent	Purified inactivated viruses (DEN 1–4), Aluminium hydroxide AS01, AS03 or AS04 adjuvants	Walter Reed Army Institute of Research (WRAIR), GlaxoSmithKline and Fiocruz	*In vivo* (phase I)	Yes	(147)
TVDV 2018	Tetravalent	DNA vaccine based on prM and E protein coding sequences cloned in VR1012 plasmid and co-administered with VAXFECTIN as an adjuvant	U.S. Army Medical Research and Development Command, WRAIR, NMRC and Vical	*In vivo* (animal and phase I)	Yes	(148)
V180 2018	Tetravalent	Recombinant proteins based on prM and 80 % of E protein of DEN 1–4 combined with different adjuvants	Merck & Co.	*In vivo* (phase I)	Yes	(148)
DSV4	Tetravalent	Virus like particles expressing EDIII of DEN 1–4	International Centre for Genetic Engineering and Biotechnology	*In vivo* (animal)	No	(148)
mRNA vaccines (prME-mRNA, E80-mRNA, and NS1-mRNA)	Tetravalent	mRNA expressing human IgE and E80 protein packaged into LNP	CAS Laboratory of Molecular Virology and Immunology, Institute Pasteur of Shanghai	*In vivo* (animal)	No	(144)

## 4 Summary and outlooks for the future

Currently, dengue stands as the most pressing arboviral disease impacting countries in the tropical and subtropical regions worldwide. The conventional vaccine approaches, such as the live attenuated vaccines, recombinant subunit vaccines, inactivated virus vaccines, viral vectored vaccines, DNA, and mRNA vaccines have been developed to prevent the transmission of all dengue serotypes in humans. These strategies primarily work by fostering defense against either the DENV virions or the envelope proteins exhibited on the surface of the causative virus. Tetravalent, live attenuated, chimeric dengue vaccines efficiently induce neutralizing-antibodies in human subjects, however, they lack the ability to neutralize the DENV-2 virus. In addition, female mice vaccinated with the live attenuated vaccine DENVax intervene with the vaccination outcome of their offspring. Despite triggering a well-balanced immune response against all the serotypes, recombinant subunit vaccines develop contamination of endotoxin with improper protein folding. Adenoviral vectored vaccines offer numerous benefits, such as the ease of genetic modifications, efficiency in detecting gene replication mistakes, and a high level of protein expression. Additionally, alphavirus-vectored dengue vaccines induce a robust immune response with only one dose of neonatal immunization in mice. On the other hand, DNA vaccines are extremely stable, economical, and conveniently producible; however, low immunogenicity limits their use. In terms of vaccination tactics, developing successful animal protection has involved priming and boosting immunizations using various vaccine combinations.

Computational advances, including advances in immunoinformatics (149), can enable improved dengue vaccine design strategies. Such approaches may include improvements in the identification of antigenic sites, structural modeling of the viral proteins, molecular docking experiments, and the examination of molecular biomimicry (150–152). By examining different serotypes and predicting how likely different parts of the dengue genome are to evolve over time, vaccines of improved efficacy, tailored for more dengue viral serotypes, can be achieved (153, 154).

Apart from NS1, Other NS proteins are NS2A, NS2B, NS3, NS4A, NS4B, and NS5. It is possible to develop inhibitors of NS3 and NS5 by evaluating their activity using biochemical assays, but there is no compound that reached clinical studies. On the other hand, NS2A, NS2B, NS3, NS4A, and NS4B do not retain any enzymatic activity. Also, challenges in crystallization and dynamic nature of these proteins hinder their structural studies (155).

Given the fragmentary effectiveness of current vaccines against dengue, we strongly advocate that future vaccine development incorporates cutting-edge vaccine concepts like mRNA, NS1-based, and mosquito-based immunization with conventional vaccines, for the improvement of their safety and efficiency against dengue infection in humans while minimizing mosquito dissemination and effectively reducing DENV presence in the environment.

## Author contributions

RA: Methodology, Resources, Software, Writing – original draft. FT: Resources, Visualization, Writing – review & editing. SD: Visualization, Writing – review & editing. MAS: Validation, Writing – review & editing. IG-S: Validation, Writing – review & editing. RTJ: Validation, Visualization, Writing – review & editing, Software. SAS: Conceptualization, Project administration, Supervision, Validation, Writing – review & editing, Resources, Software, Writing – original draft.
